# Evaluation of the Versius Robotic Surgical System for Procedures in Small Cavities

**DOI:** 10.3390/children9020199

**Published:** 2022-02-03

**Authors:** Marit Kayser, Thomas Franz Krebs, Ibrahim Alkatout, Timo Kayser, Katja Reischig, Jonas Baastrup, Andreas Meinzer, Katja Ulrich, Daniar Osmonov, Robert Bergholz

**Affiliations:** 1 Department of General, Visceral, Thoracic, Transplant and Pediatric Surgery, UKSH University Hospital of Schleswig-Holstein Kiel Campus, Arnold-Heller-Strasse 3, 24105 Kiel, Germany; mailmariluu@gmx.de (M.K.); thomasfranz.krebs@kispisg.ch (T.F.K.); timo_kayser@hotmail.com (T.K.); katja.reischig@uksh.de (K.R.); Jonas.baastrup@uksh.de (J.B.); andreas.meinzer@uksh.de (A.M.); 2 Department of Pediatric Surgery, Ostschweizer Children’s Hospital, Claudiusstrasse 6, 9006 St. Gallen, Switzerland; 3 Department of Obstetrics and Gynecology, UKSH University Hospital of Schleswig-Holstein Kiel Campus, Arnold-Heller-Strasse 3, 24105 Kiel, Germany; ibrahim.alkatout@uksh.de; 4 Department of Vascular Surgery, Diakonissen Hospital, Knuthstrasse 1, 24939 Flensburg, Germany; ulrichka@diako.de; 5 Department of Urology, UKSH University Hospital of Schleswig-Holstein Kiel Campus, Arnold-Heller-Strasse 3, 24105 Kiel, Germany; daniar.osmonov@uksh.de

**Keywords:** robotics, laparoscopy, computer assisted laparoscopy, pediatric surgery, CMR Versius, preclinical study

## Abstract

Background: The Versius^®^ is a recently approved robotic surgical system for general surgery procedures in adults. Before any application in children, data of its feasibility and safety in small cavities has to be compiled, beginning with inanimate models. Therefore, the aim of this preclinical study was to assess the Versius^®^ system for its performance in small boxes simulating small body cavities. Methods: In total, 8 cardboard boxes of decreasing volumes (15.75 L to 106 mL) were used. The procedures, two single stitches with two square knots each, were performed in every box, starting in the largest and consecutively exchanging the box to the next smaller one. The evaluation included procedure time, port placement and pivot point setup, arrangement of the robotic arms and instrumentation, amount of internal and external instrument–instrument collisions and instrument–box collisions. Results: All procedures could be successfully performed in all boxes. The procedure time decreased due to the learning curve in the first four boxes (15.75 to 1.87 L) and consecutively increased from boxes of 1.22 L up to the smallest box with the dimensions of 4.4 × 4.9 × 4.9 cm^3^. This may be based on the progress of complexity of the procedures in small cavities, which is also depicted by the synchronous increase of the internal instrument–instrument and instrument–box collisions. Conclusion: With the use of the Versius^®^ robotic surgical system, we were able to perform robotic reconstructive procedures, such as intracorporal suturing and knot tying, in cavities as small as 106 mL. Whether this system is comparable or even superior to conventional laparoscopic surgery in small cavities, such as in children, has to be evaluated. Furthermore, before any application in newborns or infants, ongoing evaluation of this system should be performed in a live animal model.

## 1. Introduction

With the magnification of the operative field, the application of 3D technology with spatial vision, improved ergonomics for the surgeon, and a greater range of motion of the robotic wristed and angulated instruments compared to traditional laparoscopic instruments, robotic-assisted minimally invasive surgery appears to be beneficial over conventional minimal invasive surgery, especially for complex reconstructive tasks, such as intracorporal suturing [1,2,3,4].

However, because of the relatively large dimensions of the wristed instruments of current robotic systems (8 mm in diameter or 5 mm but with longer distal jaws due to mechanical restrictions), these surgical robotic systems are rarely used in smaller children, such as infants and newborns [5,6,7,8].

With the introduction of the Versius^®^ robotic system (CMR Surgical), certified for abdominal surgical procedures in adults, which offers wristed instruments measuring five millimeters in diameter with shorter articulating jaws, robotic interventions in infants and newborns appear achievable. Currently, no data exists concerning the technical feasibility of the Versius^®^ robotic system in small cavities. Thus, the aim of this study was to test the Versius^®^ system on its ability to perform complex reconstructive surgical tasks, best replicated by intracorporal suturing and knot tying, in small boxes simulating pediatric-sized cavities [9,10,11,12,13].

## 2. Materials and Methods

This study was conducted in the Quincke Research Building at the University Medical Center (UKSH, Kiel Campus) of the Christian-Albrechts-University of Kiel (CAU). Institutional review board approval was obtained from the local ethics committee of the UKSH and CAU (reference D562/21), and no animals or patients were included in this study as an inanimate model with cardboard boxes was used.

**Figure 1:**
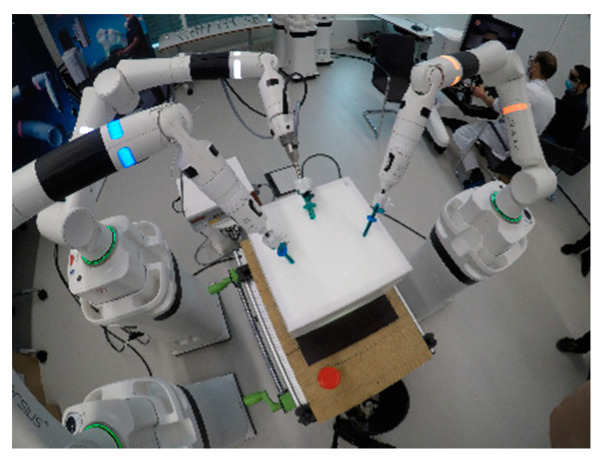
The Versius system setup with the 10 mm 3D 0° camera and 5 mm articulated needle-drivers for the right and left hand of the surgeon seen from above in a bigger box. The surgeon’s console is seen on the right. The ports, which were only used in the first two large boxes, were the Kii Sleeve with Advanced Fixation and Optical Access (Applied Medical 5 × 150 mm, 12 × 150 mm). The insertional depth was around 6 cm.

**Figure 2:**
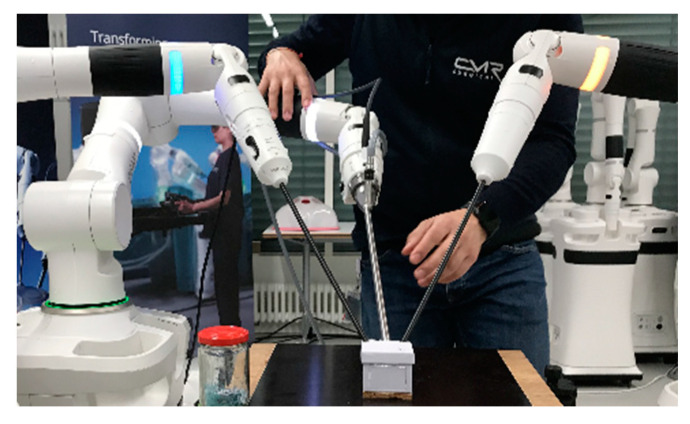
The Versius system setup with the 10 mm 3D 0° camera and 5 mm articulated needle-drivers for the right and left hand of the surgeon. The camera and instruments were used without ports in the small box, and the pivot point was set manually on the instrument shaft outside of the box margin.

A Versius^®^ robotic-system was supported by a research grant of CMR. The system consists of a master console and 3 single arms, each on its own base, which operate either a 3D 10 mm 0° or 30° camera or 5 mm articulated needle-drivers (Figure 1 and Figure 2).

**Figure 3:**
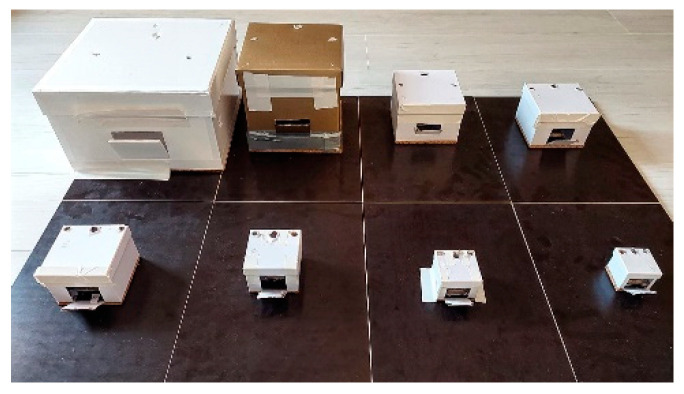
The eight cardboard boxes with decreasing volume were fastened onto a wooden plate. A Trapdoor was cut into the anterior surface for faster exchange of the sutures and markers.

Eight custom made cardboard boxes with decreasing volume (Table 1, Figure 3) were used as simulated pediatric cavities. The boxes were screwed onto a wooden plate, which was fastened on a standard work bench (Figure 2).

**Figure 4:**
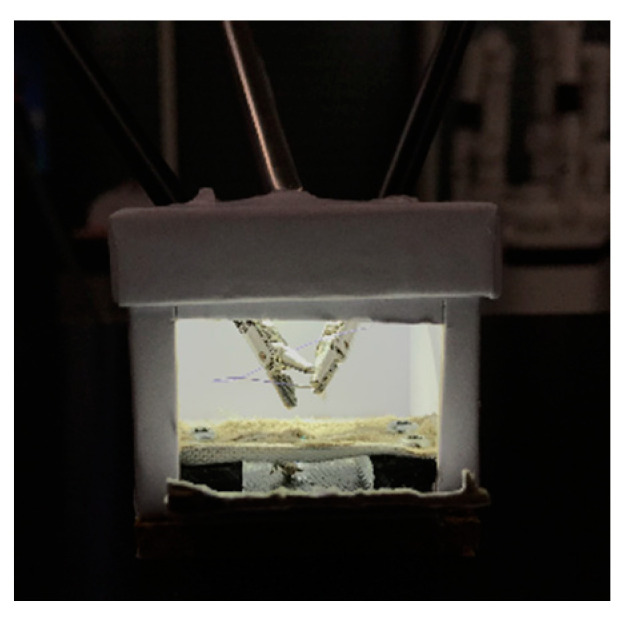
Through the trapdoor, the 3D-printed base (black) can be seen with the leather patch on top of it.

A leather patch was attached in every box on a custom 3D-printed plastic base, which was screwed onto the box floor. The leather patch was slit in the middle for 2 cm to simulate the tissue that had to be sutured together (Figure 4).

The surgical procedures that had to be performed inside the boxes consisted of suturing two single stitches, each followed by two consecutive intracorporal square knots to approximate the slit leather patch. The sutures (Ethibond 3-0 V5 or Vicryl 5-0 TF-1; Ethicon, Johnson and Johnson, Neuss, Germany), the position of the leather patch inside the box, and the triangulation of the instruments were adapted according to the available operative space in each box.

Seven participants (a Versius^®^ experienced adult gynecologic surgeon and six Versius^®^ inexperienced pediatric or vascular surgeons, pediatricians, and general practitioners) were included by personal communication of the Kurt Semm Center of the University Medical Center Campus Kiel and every participant consented to the study, its evaluation, publication, and co-authorship of this manuscript.

The exercises were performed in one session without splitting between several days. The procedures were started in the largest box. After their completion, the box was exchanged with the next smaller one to gradually decrease the volume of the simulated cavity. All procedures were recorded for later blinded analysis. Outcome parameters were completion of the task (yes, no), operating time (seconds), rate of internal instrument–instrument or instrument–box collisions (total collisions/total amount of knots per each box), rate of external instrument–instrument collisions (total collisions/total amount of knots per each box), distance between the instruments and camera ports in centimeters (Table 1), and any breaking of the suture, needle, or a knot differing from a surgical square knot [1].

The operating time was plotted against the decreasing volume of the boxes on the X-axis. This enabled two observations: first, the operating time could be analyzed for the decreasing volumes of the simulated cavities, and second, the anticipated learning curve of the surgeons by increasing the operating load with the Versius^®^ system could be examined. The Mann–Whitney U test was used to test for differences in skewed data with SPSS Version 20.0 for Mac.

## 3. Results

The procedures could be performed by all participants in all eight boxes.

The robotic camera and instrument placement was adapted to the size of the cardboard boxes and port triangulation decreased from 13–12.5–19 cm to 2.5–2.4–4.5 cm (camera to left and right instrument and instrument to instrument, Table 1).

The collision of instruments increased within smaller cavities but did not impede the completion of the procedures in any of the boxes (Table 2).

### 3.1. Instrumentation and Pivot Point Work-Around for Small Cavities

The calibration of the pivot point on each robotic arm, which should be at the level of the abdominal fascia, was performed after insertion of the instrument by autonomous small rotational movements of the system while measuring the forces on the instrument shaft to calculate the optimal pivot point. As specified by the manufacturer, the robotic instruments should be inserted through laparoscopic ports and with a minimal insertion depth of 5 cm for the abovementioned calibration procedure. Therefore, the pivot point cannot be adjusted more distally towards the tip of the instrument than 5 cm. This manufacturer-given standardization of the minimal insertion depth of the robotic instruments hindered procedures in cavities less than 6.2 cm (box 6). We therefore had to adopt an off-label work-around solution to perform the procedures in smaller-sized boxes. First, instruments were inserted directly into the boxes without using operative ports, analogous to stab incisions routinely used in pediatric laparoscopy to reduce the space needed for triangulation (Figure 2 and Figure 4) [2]. Second, the pivot point was set manually by firmly holding the instrument during calibration at a spot on the instrument shaft, which was outside of the box and therefore more than five centimeters proximal to its tip as required by the system.

### 3.2. Procedure Time and Collisions

**Figure 5:**
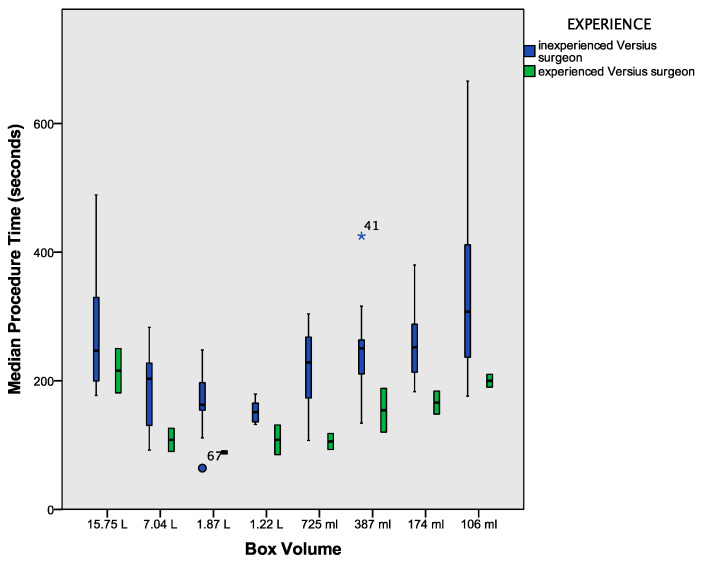
Boxplot of the procedure time (y-axis) plotted against the boxes beginning in the largest box with descending size (x-axis). The participants started in the largest box, thus the number of procedures performed per participant increased as the size of the boxes decreased. The outliers all belong to the group of Versius^®^ inexperienced participants. * and the dot denote the outliers internal database reference number.

The procedure time of both the experienced and inexperienced participants decreased gradually in the first four boxes, and an increase in the procedure time was observed starting in the fifth box (Table 2, Figure 5).

The procedure time in the largest and smallest box did not show a significant difference in either group (*p* = 0.734 in the inexperienced group, *p* = 1 in the experienced participant, and *p* = 0.843 for all participants).

The increase in the rate of collisions during procedures in smaller boxes is displayed in Table 2.

## 4. Discussion

We were able to demonstrate that surgical reconstructive procedures, such as suturing and knot tying, are feasible using the Versius^®^ robotic system in cavities with small volumes, such as 106 mL. The increasing technical challenge and rate of collisions of the instruments did not hinder the participants from completing all procedures. This is also reflected by the operating time, which was not significantly longer between the biggest and smallest box (Figure 5) and implied an advancement in experience operating with the Versius^®^ system. Simultaneously, decreasing the size of the boxes generated more boxes and thus more procedures performed by each participant and demonstrated the learning curve with the Versius^®^ system, which corresponds to earlier reported learning curves with this system [14].

The increase of the procedure time in smaller boxes, impeding the learning curve, may be due to the increased complexity of the procedures in small cavities, also reflected by the increased rate of collisions. Similar effects of multiphasic learning curves have been described for robotic adult surgery with the da Vinci^®^ system and the Senhance^®^ system by our group [1,6].

As specified by the manufacturer, the minimal insertion depth of the instruments of 5 cm into the patient’s abdomen or thorax for calibration of the pivot point limits the size of the cavities operated in. Applying a work-around with hitherto new and yet untried methods (robotic instrument insertion via stab incisions without ports and the extracorporeal manual calibration of the pivot point) made it possible to perform the procedure with the Versius^®^ system in cavities even smaller than 5 cm in diameter. Whether this work-around is safe in human application has to be evaluated in live animal models. As the calculation of the pivot point is performed by software, reprogramming the system for an alternate pivot point calculation algorithm may also help in smaller cavities.

### 4.1. Comparison of the Versius^®^ System to the Senhance^®^ and da Vinci^®^

Comparable data of the robotic systems currently approved for surgery in children, the da Vinci^®^ (Intuitive Surgical, Sunnyvale, CA, USA, since 2001) and Senhance^®^ (Asensus Surgical, former Transenterix, Durham, NC, USA, since 2020), can be found. A study with the da Vinci^®^ system showed significant instrument–instrument or instrument–box collisions within boxes measuring 4.0 and 4.5 cm (64 and 91 mL, respectively), preventing surgeons from performing the suturing procedures although the system appeared feasible in a small pig model for robotic fundoplication [7,15,16,17]. Data on the Senhance^®^ system appear more encouraging, as suturing procedures were possible in boxes as small as 90 mL and in animal models of less than 7 kg body weight [1,18].

Compared with the da Vinci^®^, the Versius^®^ system also provides 5 mm diameter instruments with an articulated tip. One may assume that both systems may be similarly effective in small cavities, but data has conversely shown that the 5 mm da Vinci instruments were less effective due to a space-consuming effect [7]. This can be explained by the shorter angulated tip and jaws of the Versius^®^ system, which make the instruments more mobile in confined spaces [8].

In contrast, the Senhance^®^ offers 3 mm instruments, which offer greater freedom of motion in small cavities, but these instruments do not offer articulating tips and jaws compared to the Versius^®^ and da Vinci^®^. These straight instruments therefore do not enable robotic “wrist like” manipulation with seven degrees of freedom, which may be a disadvantage concerning complex reconstructive procedures in small cavities compared to competing robotic systems but not to conventional laparoscopy.

The instruments of the Versius^®^ can be inserted directly without ports, which allow for the abovementioned work-around for the pivot point calibration, similar to our technique with the Senhance^®^ system [1,18]. The da Vinci^®^ requires its own operative ports with a specified insertion length for docking and operating the system. Their depth of insertion can be varied by the surgeon, but the pivot point is fixed and cannot be changed, making this technique somewhat less flexible [3,4].

Another difference between the Versius^®^ and the da Vinci^®^ and comparable to the Senhance^®^ is the technical setup of the robot: the Versius^®^ and Senhance^®^ have three separate bases with a single robotic arm attached for the instrument or camera. Thus, every base can be placed separately in the room, adapting to the individual required setup for each surgical procedure. Whether this has an effect on the surgical procedures in infants and neonates has to be evaluated.

### 4.2. Limitations

Although we were able to demonstrate that robotic procedures can be performed in small cavities simulating neonates, there are some limitations to be discussed. First, one may argue that the volumes that have been tested do not represent operative spaces in pediatric surgery. Measuring the abdominal or thoracic volumes in infants is seldom possible, but in 1989, Chapman reported the lung volumes of infants measured with echo planar imaging of around 110–200 mL for one side of the thorax, which corresponds with our models [19]. Another point to discuss is whether any data on the limits of movement in small spaces was available prior to the product launch of the Versius^®^ system. As we do not have any insight into the research and development department of CMR Surgical, we can only speculate on whether any data was generated on the limits of the robot in small spaces, be it in models or from virtual simulation. We assume that the main market for surgical robotic companies is surgery on “grown ups”, especially urology, gynecology, and general surgery. In adult surgery, the abdominal cavity is rarely so small that any limitation of operative space my play an important role, in contrast to pediatric surgery. As surgical robots are thus primarily designed for procedures on adults, we speculate that any limitation of small spaces has not crossed the minds of the developers and has therefore not been evaluated yet, especially concerning pediatric patients.

Second, the expertise of the participants might have had an impact on the procedures. One might argue that well-trained Versius^®^ surgeons will perform better than untrained general physicians or pediatricians. This study was conducted to evaluate whether robotic procedures are feasible in small cavities with the Versius^®^ system. All participants were able to complete the procedures even in the smallest cavity, therefore the primary aim of this study was reached and even superior trained Versius^®^ surgeons will not be able to perform better than the already accomplished aim. Although, their median procedure time might be faster, which was demonstrated by comparing the Versius^®^ experienced and Versius^®^ inexperienced participants. However, the increased procedure time did not impede the completion of the procedures.

One can argue that including non-surgeons in the group of Versius^®^ inexperienced participants might act as a confounder. However, the primary aim of our study was to evaluate the technical feasibility of the Versius^®^ in small spaces. As this technical feasibility of the robotic system should be independent of the surgeon’s expertise, we tried to include a wide range of not at all to less or more trained laparoscopic and one Versius experienced surgeon who underwent special training with this system prior to our study. Furthermore, despite including non-surgeons into the group of Versius^®^ inexperienced participants, this group still displayed a learning curve and a procedure time that was not significantly different between the smallest and the largest box.

Concerning the issue of gas insufflation, for which a port is required, at least one will have to be used in small patients when using the Versius^®^ instruments without ports. As the Versius^®^ can be used with any ports available, similar to the Senhance^®^ and in contrast to the da Vinci^®^, which requires its own ports for docking the system, we suggest applying a reusable 5 mm metal port for the camera and gas insufflation. The insertion depth of 5 cm is needed for the robotic instruments while the port insertion may be shorter. We are planning to evaluate this issue in a live animal model.

The procedures were completed in the small boxes by applying a work-around by inserting the instruments directly without ports and by manually setting the pivot point on the instrument shaft outside of the simulated abdominal wall. The pivot point on the instrument shaft being inside the abdominal wall is essential to reduce shear forces on the abdominal or thoracic wall while moving the instruments. Applying this technique in a human newborn or infant might result in excessive force on the abdominal wall and result in damage to the young patient. Therefore, before any application of this technique in humans, animal studies have to be performed with an additional focus on safety. This stepwise approach to evaluating new robotic systems, from inanimate to animate models and then comparative studies of the different techniques and open surgery, was proposed by us in an earlier report [18].

## 5. Conclusions

We were able to perform robotic intracorporal suturing and knot tying in simulated neonatal cavities as small as 106 mL with the Versius^®^ robotic system. Any probable application of the Versius^®^ system in children has to be preceded by studies in animal models, comparing the system to conventional three-port laparoscopic or thoracic pediatric surgery.

## Figures and Tables

**Table 1:** Size of the cardboard boxes, the calculated volume, ΔLC: distance of the left instrument to the camera, ΔRC: distance of the right instrument to the camera, ΔLR: distance between both instruments.

Box No.	Width (cm)	Height (cm)	Depth (cm)	Volume *	ΔLC (cm)	ΔRC (cm)	ΔLR (cm)
1	17.5	30	30	15.75 L	13	12.5	19
2	18.5	19.5	19.5	7.04 L	10	11.5	17
3	13.9	13.9	9.7	1.87 L	8	8	12.5
4	8.6	11.9	11.9	1.22 L	5.5	5.5	11
5	7.4	9.9	9.9	725 mL	4.5	4.5	9
6	6.2	7.9	7.9	387 mL	4	3.5	7
7	5	5.9	5.9	174 mL	2.5	2.5	5
8	4.4	4.9	4.9	106 mL	2.5	2.4	4.5

* Volume is given in liters (L) or milliliters (mL).

**Table 2:** Procedure times and collisions by each group in every box.

	Box	Median Procedure Time (Sec)	Shortest Procedure Time (Sec)	Longest Procedure Time (Sec)	Internal Instrument/Instrument Collisions per Knot	Internal Instrument/Box Collisions per Knot	External Instrument/Instrument Collisions per Knot
**Versius inexperienced Surgeons (*n* = 6)**	**1**	264.5	177.0	489.0	5.42	0.42	0
	**2**	203	92.0	283.0	5.25	0.75	0
	**3**	162.5	64.0	248.0	4.4	0.2	0
	**4**	151	132.0	179.0	4.7	0.8	0
	**5**	228.5	107.0	304.0	7.11	0.78	0.44
	**6**	250	134.0	425.0	5.82	0.55	0.73
	**7**	252	183.0	380.0	4.17	1.33	1.17
	**8**	307	176.0	666.0	7.36	2.55	1.82
**Versius experienced Surgeon (*n* = 1)**	**1**	215.5	181.0	250.0	0	0	0
	**2**	108.0	90.0	126.0	1.50	0	0
	**3**	88.5	86.0	91.0	0	0	0
	**4**	108.0	85.0	131.0	1	0	0
	**5**	105.5	93.0	118.0	1	1.50	0
	**6**	154.0	120.0	188.0	2.50	1	0
	**7**	166.0	148.0	184.0	0.50	0	2.5
	**8**	200.0	190.0	210.0	0.50	1	1

## Data Availability

Data presented is contained within this article.
